# Validation of a three-item Fatigue Severity Scale for patients with substance use disorder: a cohort study from Norway for the period 2016–2020

**DOI:** 10.1186/s12955-021-01708-w

**Published:** 2021-03-02

**Authors:** Jørn Henrik Vold, Rolf Gjestad, Christer F. Aas, Eivind Meland, Kjell Arne Johansson, Lars Thore Fadnes, for the INTRO-HCV Study Group

**Affiliations:** 1 Department of Addiction Medicine, Haukeland University Hospital, Bergen, Norway; 2 Department of Global Public Health and Primary Care, University of Bergen, Bergen, Norway; 3 Division of Psychiatry, Research Department, Haukeland University Hospital, Bergen, Norway

**Keywords:** Substance-related disorders, Fatigue, Quality of life, Validation, Fatigue Severity Scale, Visual Analogue Fatigue Scale, Activities of daily living

## Abstract

**Background:**

Little attention has been paid to customising fatigue questionnaires for patients with Substance Use Disorders (SUDs). The present study aims to validate and shorten the nine-item Fatigue Severity Scale (FSS-9) and Visual Analogue Fatigue Scale (VAFS) for use with this population.

**Methods:**

We used data from a nested cohort with annual health assessments with responses on the FSS-9 and VAFS. During the period 2016–2020, 917 health assessments were collected from 655 patients with SUD in Bergen and Stavanger, Norway. A total of 225 patients answered the health assessment at least twice. We defined baseline as the first annual health assessment when the health assessments were sorted chronologically per patient. We checked for internal consistency, and we used longitudinal confirmatory factor analysis (CFA) and linear mixed model (LMM) analysis to validate and shorten the FSS-9 and VAFS.

**Results:**

The internal consistency of the FSS-9 was excellent with a Cronbach’s α of 0.94 at baseline and 0.93 at the second annual health assessment. When shortening the FSS-9 to a three-item FSS (FSS-3, items 5–7), the Cronbach’s α was 0.87 at baseline and 0.84 at the second health assessment. The internal consistency was not affected when the VAFS was added to the FSS-3 and the FSS-9. The longitudinal CFA model showed a well-fitting model for the FSS-3 (χ^2^ = 13.33, degree of freedom = 8, *P* = 0.101). The LMM analysis showed equal linear changes at the individual level for the FSS-3 (slope: 0.00, *P* > 0.05) and FSS-9 (slope: 0.01, *P* > 0.05) between the health assessments.

**Conclusion:**

The FSS-9 could be shortened to the FSS-3 with high validity and reliability for patients with SUDs and the addition of VAFS did not provide much added variability.

## Background

Fatigue is a common subjective health complaint that significantly affects patients with chronic diseases [1–3]. For patients with substance use disorders (SUDs), fatigue is associated with underlying physical and mental health problems—such as hepatitis C virus infection, depression, and an increased risk of suicide [4–6]. However, we have scarce evidence about fatigue in the SUD population, and validated questionnaires for surveying fatigue and investigating how it varies over time do not exist for this patient group. To ensure high-quality clinical trials focusing on fatigue, developing a validated and customized fatigue questionnaire aimed for SUD patients is therefore of particular interest.

Patients with SUDs often live in a chaotic situation with extensive medical and psychosocial health challenges, including polysubstance use, substance intoxications and withdrawals, psychiatric comorbidities (e.g., attention deficit hyperactivity disorder, psychosis disorders, or personality disorders), chronic viral hepatitis, financial risk, and temporary living situations [7, 8]. This might make surveying fatigue with questionnaires particularly challenging and might influence the patients’ fatigue experiences and how they respond to questionnaires [9–11]. Using simple wording and phrases, avoiding the use of questions which differ in subtle ways which make them appear repetitive, and administering measures that produce reliable and valid scores based on very few questions, might be essential to ensuring reliable results in clinical trials on fatigue in this population.

The nine-item Fatigue Severity Scale (FSS-9) and the Visual Analog Fatigue Scale (VAFS) are two well-known fatigue questionnaires. The evidence for the validity of the FSS-9 has been obtained for a wide range of chronic infectious and neurological diseases—such as hepatitis C virus infection [1], Parkinson’s disease [12], myasthenia gravis [2], systemic lupus erythematosus [13], and stroke [3]; while the VAFS has shown evidence for a good validity and reliability in post-stroke patients [14]. In two validation studies, the VAFS was used alongside the FSS-9 with high correlations [15, 16]. Furthermore, excellent validity and reliability were achieved when the FSS-9 was shortened into a seven-item version [17, 18], while uncertain results were reported when a five-item version was validated [19].

With this validation study we sought to address two gaps in the literature on the assessment of fatigue in patients with SUDs: (1) to evaluate whether the scores produced by a shortened version of FSS-9 can be interpreted similarly to the scores produced by the FSS-9, and (2) to determine whether there is value to adding the VAFS to either the FSS-9 or the shortened version of FSS-9.

We hypothesised that the FSS-9 and VAFS questionnaires can create a short version of the FSS-9 which demonstrates strong evidence of reliability and validity when used with patients who have SUDs.

## Methods

### Data source

We drew data from a nested cohort from the INTRO-HCV trial that collected data on patients with SUDs in Bergen and Stavanger, Norway [20]. We recruited patients receiving opioid agonist therapy in Bergen and Stavanger and patients with SUDs receiving healthcare from the primary health clinics in the municipality of Bergen. This study included all patients in the cohort who had answered the FSS-9 and/or VAFS in the study period from May 2016 to January 2020.

### Data collections

All included patients were invited to an annual health assessment, including FSS-9 and VAFS measurements and a survey of their current sociodemographic situation. We collected all data in a health register using data collection software (Checkware^®^) under the supervision of research nurses.

### Study sample

We conducted 917 health assessments of 655 patients, and this included 916 FSS-9 measurements and 915 VAFS measurements during the study period. We defined a measurement as when at least one of the items in the FSS-9 or the VAFS were answered during a health assessment. Baseline was defined as the first health assessment including measurements of the FSS-9 or VAFS when the health assessments were listed chronologically. The FSS-9 and VAFS were completely answered in 914 health assessments. For the remaining three health assessments, one patient only completed the VAFS and not the FSS-9, one only answered five of the nine items on the FSS-9 and did not complete the VAFS, and a third completed the FSS-9 and not the VAFS. Of the 655 included patients, 188 completed two health assessments, while 37 patients completed three health assessments. The time intervals between the annual health assessments varied with a mean of 12 months (standard deviation (SD): 4 months) (Additional file 1). Due to the relatively small number of patients with three health assessments, we used two health assessments when estimating internal consistency reliability and construct validity. For patients with three annual health assessments, we only included the first (baseline) and second health assessments in these analyses.

### Measuring fatigue

We used the FSS-9 and VAFS to measure the level of fatigue. The FSS-9 measures fatigue during the past week, and it includes items regarding: mental and physical functioning, motivation, exercise, carrying out certain duties, and interference with work, family, or social life. The VAFS measures the patient’s general experience of fatigue. The FSS-9 was answered on a Likert scale from 1 (no fatigue) to 7 (worst fatigue) and the VAFS was answered by placing a mark on a line from 0 (no fatigue) to 10 (worst fatigue) that represent the fatigue level. The data collection software only allowed valid responses for each question and prompted for responses to unanswered questions before submission in order to minimise missing data. In a previous study, the US-English version of the FSS-9 has been translated into Norwegian by a qualified native Norwegian-speaking translator and back-translated into US-English by an authorised native US-English-speaking translator (Additional file 2) [21].

### Statistical analysis

We used Stata/SE 16.0 (StataCorp, TX, USA) for descriptive analysis and IBM SPSS version 24.0 and M*plus* version 8.4 for reliability analysis (Cronbach’s α if-item-deleted and Item-Total correlation), for confirmatory factor analysis (CFA), and for linear mixed model (LMM) analysis (M*plus*: TwoLevel analysis). The threshold for statistical significance was set to *P* < 0.05 for all analyses unless otherwise stated.

#### Internal consistency of the FSS-9 and the shortened version of FSS-9 (FSS-3), and these scales including the VAFS

We calculated the internal consistency of the FSS-9 and this scale including the VAFS at baseline and at the second health assessment. As a part of the validation study, we explored whether there was value in adding the VAFS to the FSS-9 by evaluating if the VAFS added more information than captured by the FSS-9. Cronbach’s α was considered to show good internal consistency if Cronbach’s α was above 0.70 [22–24]. We then shortened the FSS-9 by deleting the item that resulted in the highest Cronbach’s α value for the remaining items (alpha-if-item-deleted analysis). The remaining items’ Cronbach’s α coefficients were recalculated, and the next item was deleted. If the remaining scale showed almost equal Cronbach’s α values after removing one or another item, clinical experience was used in the decision of what item we removed. We deleted items that were less adaptable to patients with SUDs, for example, items about employment (unemployment was common in this population) and items with complex phrases and wordings that could be difficult to understand for patients with SUDs when they were intoxicated or went through substance withdrawals. Furthermore, we calculated Cronbach’s α for the VAFS plus the shortened version of FSS-9 (FSS-3) at baseline and at the second health assessment. Due to strong inter-item correlations and good reliability in previous studies evaluating the FSS-9 alongside the VAFS [15, 16], we expected that the VAFS would not provide much added variability than that captured by the FSS-9 and FSS-3 questionnaires at baseline and at the second health assessment.

#### Longitudinal confirmatory factor analysis for evaluating the fit of the FSS-3 and FSS-9, and these scales including the VAFS

We used CFA models to test the structure of the items in the FSS-3 and FSS-9, and these scales including the VAFS at baseline and at the second health assessment in order to evaluate the relationships between the items and their underlying latent factors [22, 25–28]. We expected that both the FSS-3 and FSS-9 should support one-dimensional models. The VAFS was added to the FSS-3 and FSS-9 to examine whether the VAFS provided more added variability in fatigue than captured by the FSS-3/FSS-9. This should be indicated by a less than a perfect correlation between the FSS-3/FSS-9 and VAFS. Further, we used longitudinal CFA in order to test measurement invariance for the FSS-3. First, we estimated a free model with all unique parameter values. We then tested for constraints in the model by setting the factor loadings within each item equal to each other at baseline and at the second health assessment. Third, we tested for equality within the residuals over time. The last model constrained the intercept values for the indicators. We used the Wald test to compare model restrictions. All CFA models were evaluated with standard fit measures: χ^2^, degrees of freedom, *p *values, Comparative fit index, Tucker Lewis Index, Root Mean Square Error of Approximation with 90% confidence interval, and the probability of close fit. A well-fitted model should have a statistically non-significant χ^2^, values of Comparative fit index and Tucker Lewis Index should be above 0.95, and Root Mean Square Error of Approximation should preferably be below 0.05 (close fit) [26]. Root Mean Square Error of Approximation above 0.10 is considered to be a poorly fitted model [26]. We used the modification index to explore model improvements if the goodness of fit measures indicated a poorly fitted model (χ^2^ difference test). We analysed all variables as continuous variables due to the relatively high number of categories in the ordinal variables (FSS-9 items ranged from 1 to 7, and the VAFS ranged from 0 to 10). The CFAs were run using the Robust Maximum Likelihood estimator. According to previous studies showing good reliability and strong inter-item correlations of the FSS-9 and this scale including the VAFS [15, 16], we expected increased support for the FSS-3 reflecting fatigue as one dimension with stronger levels and homogeneity in the factor loadings than for the FSS-9, also when including the VAFS at baseline and at the second health assessment. This should be the consequence of reducing the scale by using the most relevant measurement indicators in this population of respondents. Furthermore, we expected that the longitudinal CFA would support measurement invariance over time for the FSS-3 [26]. Measurement invariance is indicated if each measurement indicator is equally important for the underlying factor over time, with equal factor loadings and intercepts within each item over time. In addition, strict invariance would be supported if residuals for each item are equal over time.

#### Linear mixed model analysis for evaluating changes in the FSS-3 and FSS-9 sum scores and the VAFS score

We used a LMM analysis (M*plus* multilevel modelling: TwoLevel) to evaluate linear changes from baseline in the sum scores of the FSS-3 and FSS-9, the scores in the separate FSS-9 items, and the VAFS score. We included all 917 health assessments. First, we estimated a full random intercept random slope model, which gave us the mean and individual variance in terms of both level and change together with the relationship between level and change [29]. We re-estimated the model as a random intercept fixed slope model if the covariance between the intercept and the slope variance was statistically non-significant. We used the M*plus* Maximum Likelihood Robust estimator to correct standard errors for potential deviation from normality [30]. In addition, interclass correlations were estimated. We used full information maximum likelihood in order to use all available measurements. The full information maximum likelihood assumes ‘missing at random’ [31]. Based on separate variables as indicators of the same underlying construction, we expected similar changes over time in the indicators and as seen in the FSS-9 sum score.

### Ethics approval and consent to participate

The study was reviewed and approved by the Regional Ethical Committee for Health Research (REC) West, Norway (reference number: 2017/51/REK Vest, dated 29.03.2017/20.04.2017). Each patient provided written informed consent prior to enrolling in the study.

## Results

### Patient characteristics at baseline

Seventy-one percent were males, and the mean age was 43 years (Table 1). Half had more than primary school as the highest level of education. Eighty-three percent received opioid agonist therapy, and 42% had injected substances in the last 30 days leading up to the health assessment. The mean values of the FSS-9 items varied from 4.43 to 5.38 at baseline (Table 2). For the VAFS, the mean value was 5.19 at baseline. The FSS-9 and VAFS variables were slightly left-skewed (skewness ranged from − 1.14 to − 0.29) and tended towards a flattened distribution (kurtosis ranged from − 1.39 to − 0.09).

**Table 1:** Baseline characteristics of patients [numbers (n) and percentages (%)]

	*N* = 655
Age (years), n (%)	
18–29	81 (12)
30–39	185 (28)
40–49	207 (32)
50–59	147 (22)
≥ 60	35 (5)
Mean (SD)	43 (11)
Gender, n (%)	
Male	462 (71)
Female	193 (29)
Highest educational level, n (%)	
Not completed primary school	40 (6)
Completed primary school (9 years)	286 (44)
Completed high school (12 years)	260 (40)
≤ 3 years of college or university	57 (9)
> 3 years of college or university	12 (2)
Receiving OAT, n (%)	542 (83)
OAT opioid, n (%):	
Methadone	212 (39)
Buprenorphine^a^	323 (60)
Long-acting morphine sulfate	7 (1)
Housing situation the past 30 days, *n* (%)	
Owned or rented housing	518 (79)
Temporary residence (homeless shelter, with family or friends)	156 (24)
Living on street	27 (4)
Prison	7 (1)
Other	27 (4)
Ever injected substances, *n* (%)	573 (94)
Injected substances the past 30 days, *n* (%)	257 (42)

*OAT* opioid agonist therapy, *SD* standard deviation

^a^“Buprenorphine” includes buprenorphine, buprenorphine-naloxone, and buprenorphine depot

**Table 2:** Descriptive information of the Nine-item Fatigue Severity Scale (FSS-9) and Visual Analog Fatigue Scale (VAFS) at baseline (FSS-9: N = 654, VAFS: N = 655)

	Mean	SD		Median		IQR	Skewness (SE)	Kurtosis (SE)	Variance
*FSS-9 items*									
I1: My motivation is lower when I am fatigued	5.38	2.04		6.00	5.00–7.00		− 1.14 (0.10)	− 0.09 (0.19)	4.16
I2: Exercise brings on my fatigue	4.73	2.12		5.00	3.00–7.00		− 0.54 (0.10)	− 1.11 (0.19)	4.49
I3: I am easily fatigued	4.54	2.15		5.00	3.00–7.00		− 0.42 (0.10)	− 1.24 (0.19)	4.60
I4: Fatigue interferes with my physical functioning	4.95	2.07		6.00	3.00–7.00		− 0.74 (0.10)	− 0.81 (0.19)	4.27
**I5: Fatigue causes frequent problems for me**	**4.43**	**2.22**		**5.00**	**2.00–7.00**		**− 0.31 (0.10)**	**− 1.39 (0.19)**	**4.91**
**I6: My fatigue prevents sustained physical functioning**	**4.62**	**2.19**		**5.00**	**2.00–7.00**		**− 0.48 (0.10)**	**− 1.22 (0.19)**	**4.79**
**I7: Fatigue interferes with carrying out certain duties and responsibilities**	**5.00**	**2.14**		**6.00**	**4.00–7.00**		**− 0.84 (0.10)**	**− 0.75 (0.19)**	**4.56**
I8: Fatigue is among my three most disabling symptoms	4.64	2.34		5.00	2.00–7.00		− 0.50 (0.10)	− 1.35 (0.19)	5.48
I9: Fatigue interferes with my work, family, or social life	4.85	2.17		5.00	3.00–7.00		− 0.69 (0.10)	− 0.95 (0.19)	4.72
VAFS	5.19	2.65		5.00	3.00–7.00		− 0.29 (0.10)	− 0.78 (0.19)	7.04

This table displays descriptive information of the FSS-9 and VAFS scores at baseline. The FSS-9 was answered on a Likert scale from 1 (no fatigue) to 7 (worst fatigue). The VAFS was answered by placing a mark on a line from 0 (no fatigue) to 10 (worst fatigue). The items in bold represent the FSS-3 items.

*FSS* Fatigue Severity Scale, *I* item, *IQR* interquartile range (25–75 percentiles), *SD* standard deviation, *SE* standard error

#### Internal consistency of the FSS-3 and FSS-9, and these scales including the VAFS

The nine-item Fatigue Severity Scale’s Cronbach’s α was 0.94 at baseline and 0.93 at the second health assessment (Additional file 3). For the FSS-3, retaining items 5–7 from the FSS-9, Cronbach’s α was 0.87 at baseline and 0.84 at the second health assessment. The internal consistency was not substantially affected when we added the VAFS to the FSS-3 and FSS-9 at baseline (FSS-3 plus VAFS: α = 0.87; FSS-9 plus VAFS: α = 0.94), and at the second health assessment (FSS-3 plus VAFS: α = 0.85; FSS-9 plus VAFS: α = 0.93).

#### Longitudinal confirmatory factor analysis for evaluating the fit of the FSS-3 and FSS-9, and these scales including the VAFS

**Fig. 1:**
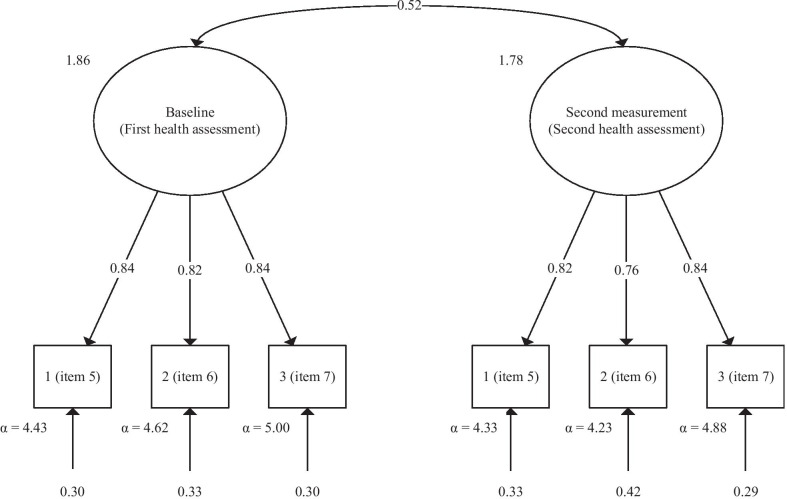
Longitudinal confirmatory factor analysis of the FSS-3 in patients with substance use disorders. FSS-3: Three-item Fatigue Severity Scale. The longitudinal factor analysis structure is shown with the upper circles representing factor 1 and the factor loadings between factor 1 and three separate question items at two different time points (baseline and second health assessment) for the three-item Fatigue Severity Scale [26]. The arrows at the bottom represent the residuals and alfa (α) values for each question item’s standardised mean values. The arrow between the circles represents the correlation (0.52) between the confirmatory factor analyses. The values of 1.86 and 1.78 on the left side of the circles represent the latent variables’ variance

The results from the CFAs comparing the fit of items in the FSS-3 and FSS-9, and these scales including the VAFS at baseline and at the second health assessment are shown in Table 3. At baseline, the unidimensional model with unique factor loadings, residuals, and intercept values resulted in a borderline fitted model, with a Comparative Fit Index and Tucker Lewis Index below the suggested levels and a Root Mean Square Error of Approximation point estimate just at the level of a poor fit for the FSS-9. The modification index showed that the estimation of the residual covariance between items 2 and 3 improved the model with the best result (Δχ^2^ = 80.1, degree of freedom (*df*) = 1, *P* < 0.001). The factor loadings for the nine items of the FSS-9 in this model were (ranged from items 1 to 9): 0.66, 0.74, 0.77, 0.78, 0.85, 0.79, 0.84, 0.84, and 0.84. The FSS-3 showed a well-fitted model. The FSS-3 and FSS-9 were highly correlated with *r* = 0.95, *P* < 0.001, giving 90% explained variance after estimating factor scores. The VAFS, together with the FSS-3 and FSS-9, respectively, gave identical results. The correlations with VAFS were *r* = 0.68, *P* < 0.001 (FSS-3) and *r* = 0.70, *P* < 0.001 (FSS-9). We obtained relatively similar results for the factor models at the second health assessment for both the FSS-3 and FSS-9 with and without the VAFS. The full FSS-9 version showed the factor loadings to be (ranged from items 1 to 9) 0.59, 0.66, 0.75, 0.69, 0.84, 0.74, 0.85, 0.80, and 0.79. The longitudinal CFA model based on the FSS-3 supported time-invariant equal factor loadings and equal residuals between the baseline and the second health assessment (Fig. 1). The correlation between the models at baseline and at the second measurement was *r* = 0.52, *P* < 0.001. A small reduction in the model fit was found if the intercept values were constrained to be equal within each measured item between health assessments. However, this simpler model was still a well-fitted model.

**Table 3:** Confirmatory factor analysis results at baseline and at the second health assessment

	χ^2^	*df*	*P*	CFI	TLI	RMSEA		
						ε_a_	ε_a 90%CI_	ε_a *p*<.05_
Time 1 (baseline)								
FSS-9	212.90	27	< 0.001	0.93	0.90	0.10	0.09, 0.12	0.00
FSS-9 ^ResCov^	132.80	26	< 0.001	0.96	0.94	0.08	0.07, 0.09	0.00
FSS-9 ^ResCov^ + VAFS	151.33	34	< 0.001	0.96	0.95	0.07	0.06, 0.09	0.00
FSS-3	0.48	2	0.788	1.00	1.00	0.00	0.00, 0.05	0.95
FSS-3 + VAFS	4.42	4	0.352	1.00	1.00	0.01	0.00, 0.06	0.87
Time 2 (second health assessment)								
FSS-9	108.14	27	< 0.001	0.90	0.86	0.12	0.10, 0.14	0.00
FSS-9 ^ResCov^	50.16	26	0.003	0.97	0.96	0.06	0.04, 0.10	0.18
FSS-9 ^ResCov^ + VAFS	65.44	34	< 0.001	0.96	0.95	0.06	0.04, 0.09	0.15
FSS-3	4.20	2	0.122	0.99	0.98	0.07	0.00, 0.17	0.27
FSS-3 + VAFS	5.37	4	0.252	1.00	0.99	0.04	0.00, 0.11	0.50
Time 1–2								
FSS-3^a^	13.33	8	0.101	0.99	0.99	0.03	0.00, 0.06	0.83
FSS-3^b^	14.47	10	0.153	1.00	0.99	0.03	0.00, 0.05	0.92
FSS-3^c^	15.48	13	0.278	1.00	1.00	0.02	0.00, 0.04	0.98
FSS-3^d^	23.35	16	0.105	0.99	0.99	0.03	0.00, 0.05	0.96

The table shows the confirmatory factor analysis results with variance and model fit of the FSS-9, FSS-9 with ResCov, FSS-9 plus VAFS, FSS-9 with ResCov plus VAFS, FSS-3, and FSS-3 plus VAFS at baseline (Time 1) and at the second health assessment (Time 2). For the FSS-3, a longitudinal confirmatory factor analysis was shown in the period from baseline to the second health assessment (Time 1–2). The mean time interval between baseline and the second health assessment was 11.6 months (SD: 4.2)

*CFI* Comparative Fit Index, *TLI* Tucker Lewis Index, *VAFS* Visual Analog Fatigue Scale, *FSS-9* Nine-item Fatigue Severity Scale, *FSS-3* Three-item Fatigue Severity Scale, ε_a_ Root Mean Square Error of Approximation (RMSEA); ε_a_ *P* < .05: Probability of close fit (RMSEA = 0.05); ResCov: Residual covariance between items 2 and 3 estimated; SD: Standard deviation

^a^Free factor loadings, intercepts, and residuals within and from baseline (time 1) to the second health assessment (time 2)

^b^Equal factor loadings from baseline (time 1) to the second health assessment (time 2): Wald test of constraints: 0.77, *P* = 0.682

^c^And equal residuals within each indicator from baseline (time 1) to the second health assessment (time 2): Wald test of constraints: 1.25, *P* = 0.682

^d^And equal intercepts within each indicator from baseline (time 1) to the second health assessment (time 2): Wald test of constraints: 8.61, *P* = 0.035

#### Linear mixed model analysis for evaluating changes in the FSS-3 and FSS-9 sum scores and the VAFS score

The linear mixed model analysis showed considerable intra-individual clustering for the FSS-3 and FSS-9 sum scores, the VAFS score, and the separate items’ scores (Table 4). However, the intraclass correlation coefficient estimated variations of 0.18 to 0.52, and these showed more variation over time in some variables than in others. The random slope and covariance between the intercept and the slope were statistically non-significant for all models. This indicated an equal linear change from baseline at the individual level. The re-estimated linear random intercept fixed slope models showed a small increase in the items 1–4, while no similar change was found in the other items. A mean change was also found in the VAFS variable.

**Table 4:** Linear mixed model analysis results for the FSS-9, FSS-3 and the Visual analog Fatigue Scale (VAFS)

	Fixed effects				Random effects								ICC
	Baseline		Change		Residual		Intercept		Slope		Cov_i,s_		
	I	*P*	S	*P*	σ^2^_e_	*P*	σ^2^_i_	*P*	σ^2^_s_	*P*	σ^2^_is_	*P*	
FSS-9^a^	4.76	***	0.01		1.46	***	1.61	***	0.00		− 0.02		0.52
FSS-3^b^	4.64	***	0.00		2.07	***	1.64	***	0.00		− 0.01		0.44
Item 1	5.35	***	0.02	*	2.52		1.53	***	0.00		− 0.04		0.38
Item 2	4.71	***	0.03	**	2.95	***	1.41	***	0.00		− 0.03		0.32
Item 3	4.51	***	0.03	**	2.40	***	2.13	***	0.00		− 0.02		0.47
Item 4	4.90	***	0.03	**	3.43	***	0.73	*	0.00		− 0.04		0.18
Item 5 ^sv^	4.38	***	0.02		2.99	***	1.84	***	0.00		− 0.02		0.38
Item 6 ^sv^	4.57	***	− 0.01		3.18	***	1.60	***	0.00		− 0.01		0.34
Item 7 ^sv^	4.97	***	0.00		2.92	***	1.62	***	0.00		− 0.02		0.36
Item 8	4.60	***	0.02		2.92	***	2.50	***	0.00		− 0.02		0.46
Item 9	4.82	***	− 0.01		2.90	***	1.90	***	0.00		− 0.00		0.40
VAFS	5.14	***	0.02	*	4.73	***	2.27	***	0.00		− 0.02		0.33

The table shows the linear mixed model analyses, including the fixed effects, the random effects, and the ICC of the FSS-3, FSS-9, VAFS, and the single items of the FSS-9. The random effects show the residual, the slope, and the covariance between the intercept and slope

Cov_i,s_, Covariance between intercept and slope; ICC: Intraclass Correlation Coefficient of between on total variance; I: Intercept (baseline); S: Slope (change); σ^2^: variance (residual, intercept and slope); σ^2^_i,s_: Covariance between intercept and slope

*P < 0.05; **P < 0.01; ***P < 0.001

^a^FSS-9: Nine-item Fatigue Severity Scale

^b^FSS-3: Three-item Fatigue Severity Scale

^sv^Items included in the FSS-3

## Discussion

The present study shows that the FSS-9 can be shortened to the FSS-3, with most of the included variance and validity retained. The value in adding the VAFS to the FSS-3 and FSS-9 did not provide much added variability. Questionnaires that are easily understood and with few items are essential to ensure high completion rates among patients with SUDs. Our findings showed that the full-scale FSS-9 had good internal reliability when tested empirically based on internal consistency. The internal consistency measured for the FSS-3 was nearly as high for the FSS-3 as FSS-9, even though it was just below a suggested 0.90 threshold [24, 32]. This supports the use of the FSS-3 as a patient-reported outcome measure in group-level comparisons, but its use in clinical practice would be controversial. The FSS-3 results from the longitudinal CFA showed a well-fitted unidimensional model with equal factor loadings and equal residuals when comparing baseline to the second health assessment. The FSS-3 and FSS-9 were almost perfectly correlated, which was in line with what we expected considering the homogeneity of the FSS-9. The factor analysis supported longitudinal stability in the indicators and confirmed the longitudinal measurement invariance. Although the fatigue level varied, the LMM analysis showed that the scoring structure was substantially stable and equal over time from baseline, which supported the validity of the indicators. This finding was somehow surprising considering the chaotic life situations and extensive substance use of many patients, which could contribute to substantial individual changes in item scores over time.

The reliability analyses showed high internal consistency for the FSS-3 and FSS-9, and the internal consistency only decreased from 0.94 to 0.87 when reducing the number of items from nine (FSS-9) to three (FSS-3). Cronbach’s α is related to the ratio between the mean covariance and the total variance, and the number of items, which makes the use of α thresholds to some degree arbitrary [22]. Our results showed that the reductions in the Cronbach’s α were accounted for by the number of items and not the covariance/total variance ratio, which is a less problematic reason for this small amount of reduction of internal consistency reliability. A homogenous and substantially equal internal consistency was also found in studies evaluating the FSS-9 in other chronic diseases such as stroke [3], hepatitis C virus infection [1], and multiple sclerosis [19], as well as studies that have shortened the Fatigue Severity Scale from nine to seven items [17, 18, 33]. In the present study, most patients were polysubstance users, of which nearly 40% had injected substances during the past 30 days. This may contribute to substantial changes in medical and psychosocial factors affecting the health assessment, for example, being affected by substances, living temporarily on the street, or having a lack of income, thus making the present highly reliable and very short FSS-3 questionnaire useful in further fatigue surveys.

The confirmatory factor analyses demonstrated unidimensional models for the FSS-9 at baseline and at the second health assessment, which was improved when adding the residual covariance between items 2 and 3. This means that the single-factor model did not fully capture these items’ responses. The explanations for this might be related to similar phrasing and wording [34], as well as the order of items 2 and 3, which might affect patients’ perception and interpretation, and increase their confusion. Moreover, the unidimensional FSS-9 factor model was in line with models reported in other studies that have validated the FSS-9 [2, 21, 33]. In those studies, however, small study samples have been a substantial limitation, contributing to a potential risk of overlooking underlying multidimensional models [2, 33]. When using a relatively large cohort of patients with SUDs, the present study showed that the unidimensional factor models of the FSS-3 and FSS-9 were maintained. Therefore, regardless of sample sizes, one can assume that the unidimensional factor models are generally well-fitted for the FSS-3 and FSS-9.

The linear mixed model analysis showed that items 5–7 included in the FSS-3 remain substantially stable and equal over time between the annual health assessments compared to the separate items 1–4 and VAFS. The result might give further arguments for the better validity of the FSS-3 questionnaire, and the FSS-3 is assumed to be less sensitive to fluctuations compared to the FSS-9. This points out that the FSS-3 might be preferred when evaluating changes in fatigue over time in the SUD population.

### Strengths and limitations

This study had some strengths. First, we collected data from patients who are difficult to reach in both research and health care. Of those, 225 patients were followed up by two or three annual health assessments, making longitudinal analyses possible. Second, patients recruited to this study answered the FSS-9 and VAFS questionnaires under different mental and psychosocial conditions, for example, when they were going through substance intoxications or withdrawal and living on the street, which might increase the generalisation of the results. The study also had some limitations. First, the majority of the patients were recruited from opioid agonist therapy, making this validation study more transferable to opioid agonist therapy populations than other substance dependence populations. Second, the FSS-9 questionnaire used was translated from US-English into Norwegian and back-translated into US-English by native Norwegian and US-English-speaking translators [21], but as far as we are aware, no specific protocol for high-quality translations was used [35]. This might slightly reduce the external validity of our results. Third, the time intervals between the baseline and the second health assessment and between the second and the third health assessment varied, which could have affected how patients scored the FSS-9 and VAFS. Forth, comparing the FSS-3 to the FSS-9, the FSS-3 might increase the risk of common method bias considering that three items are more likely to be recalled and more accessible in the short-term memory than nine items [36]. Previous studies have not evaluated the impact of common method bias; however, high reliability was achieved when validating a shortened FSS-9 into seven items in various study samples [17, 37]. Nevertheless, these studies detected cross-sample differences between items 3, 5, 6, and 9, which corresponded to two items (5 and 6) in the FSS-3 questionnaire. This points out the need for further validation and shortening studies on the FSS-3 when adapting it to other populations. Fifth, the psychometric properties of the FSS-3 are not thoroughly investigated [34, 38]. We had assessed the construct validity of the FSS-3; however, we did not know that the criterion validity is conceptually equivalent to the FSS-9, and this scale including the VAFS. For further research, we call for more longitudinal data on the SUD population to improve the validity and the psychometric properties of the FSS-3 and make it even more useful for clinicians and SUD researchers.

## Conclusion

The present study demonstrates that the FSS-9 can be shortened to just the FSS-3 among patients with SUDs. The value in adding the VAFS to the FSS-3 and FSS-9 did not provide much added variability. We found that the FSS-3 was more consistent in the structure of changes in fatigue levels compared to the FSS-9. The FSS-3 seems to be a useful patient-reported outcome measure of fatigue in this population at a group level, although the clinical relevance at an individual level remains more controversial.

## Supplementary Information

**Additional file 1.** The number of months from baseline to the second or third measurements. No.: Number of patients; SD: Standard deviation; ref.: Reference. Tables display the number of patients with one, two, and three health assessments, including the Fatigue Severity Scale and the Visual Analog Fatigue Scale measurements. The table displays the time interval between the second and third health assessments and baseline.

**Additional file 2.** The US-English and the Norwegian versions of the nine-item Fatigue Severity Scale. FSS-9: Nine-item Fatigue Severity Scale. All items in the FSS-9 are answered with a Likert scale from 1 to 7 where 1 indicates “strongly disagree” and 7 indicates “strongly agree”.

**Additional file 3.** The Cronbach’s α if-item-deleted and Item-Total correlation for the FSS-3 and FSS-9, and these scales including the VAFS. FSS-9: Nine-item Fatigue Severity Scale; FSS-3: Three-item Fatigue Severity Scale; VAFS: Visual Analogue Fatigue Scale; No.: Number of patients. *Due to high internal consistency and almost equal Cronbach’s α of the remaining items, we deleted item 8 using clinical judgement. Item 8 could be difficult to answer if patients were affected by substances or were going through substance withdrawals. **We deleted item 9 using clinical judgement due to high internal consistency and almost equal Cronbach’s α in the remaining items and the fact that unemployment was frequently reported among patients with SUDs. Tables a) and c) show the Cronbach’s α if-item-deleted for the FSS-3 and FSS-9, and these scales including the VAFS using data from the first health assessment (baseline) (Table a) and the second health assessment (Table c). Tables b) and d) show the Item-Total correlation for the FSS-3 and FSS-9, and these scales including the VAFS using data from the first health assessment (baseline) (Table b) and the second health assessment (Table d). The values in bold represent the FSS-3’s Cronbach’s α if-item-deleted and Item-Total correlation.

## Data Availability

No additional data are available due to data protection requirements.

## Abbreviations

CFA: Confirmatory Factor Analysis
FSS-3: Three-item Fatigue Severity Scale
FSS-9: Nine-item Fatigue Severity Scale
LMM: Linear mixed model
REC: The Regional Ethical Committee for Health Research
SD: Standard deviation
SUD: Substance use disorder
VAFS: Visual Analog Fatigue Scale
