# Supplementary material for: Validation of a three-item Fatigue Severity Scale for patients with substance use disorder: a cohort study from Norway for the period 2016–2020
Source: Health Qual Life Outcomes. 2021 Mar 2;19:69. doi: 10.1186/s12955-021-01708-w (PMC7923309; doi:10.1186/s12955-021-01708-w)
Supplement: Supplementary file 1 — Additional file 1. The number of months from baseline to the second or third measurements. No.: Number of patients; SD: Standard deviation; ref.: Reference. Tables display the number of patients with one, two, and three health assessments, including the Fatigue Severity Scale and the Visual Analog Fatigue Scale measurements. The table displays the time interval between the second and third health assessments and baseline. [file 12955_2021_1708_MOESM1_ESM.docx]

**Additional File 1**

Title: The number of months from baseline to second and third health assessments

| Health assessment | No. | Months | | | | |  |  |
| --- | --- | --- | --- | --- | --- | --- | --- | --- |
|  |  |  | Percentiles | | | | |  |
|  |  | Mean (SD) | | 25 | 50 | 75 | | |
| First (baseline) | 655 | 0 (ref.) | | 0 (ref.) | 0 (ref.) | 0 (ref.) | | |
| Second | 225 | 11.60 (4.17) | | 8.74 | 10.91 | 13.96 | | |
| Third | 37 | 18.60 (5.60) | | 15.63 | 18.33 | 24.31 | | |
