# Supplementary material for: Validation of a three-item Fatigue Severity Scale for patients with substance use disorder: a cohort study from Norway for the period 2016–2020
Source: Health Qual Life Outcomes. 2021 Mar 2;19:69. doi: 10.1186/s12955-021-01708-w (PMC7923309; doi:10.1186/s12955-021-01708-w)
Supplement: Supplementary file 3 — Additional file 3. The Cronbach’s α if-item-deleted and Item-Total correlation for the FSS-3 and FSS-9, and these scales including the VAFS. FSS-9: Nine-item Fatigue Severity Scale; FSS-3: Three-item Fatigue Severity Scale; VAFS: Visual Analogue Fatigue Scale; No.: Number of patients. *Due to high internal consistency and almost equal Cronbach’s α of the remaining items, we deleted item 8 using clinical judgement. Item 8 could be difficult to answer if patients were affected by substances or were going through substance withdrawals. **We deleted item 9 using clinical judgement due to high internal consistency and almost equal Cronbach’s α in the remaining items and the fact that unemployment was frequently reported among patients with SUDs. Tables a) and c) show the Cronbach’s α if-item-deleted for the FSS-3 and FSS-9, and these scales including the VAFS using data from the first health assessment (baseline) (Table a) and the second health assessment (Table c). Tables b) and d) show the Item-Total correlation for the FSS-3 and FSS-9, and these scales including the VAFS using data from the first health assessment (baseline) (Table b) and the second health assessment (Table d). The values in bold represent the FSS-3’s Cronbach’s α if-item-deleted and Item-Total correlation. [file 12955_2021_1708_MOESM3_ESM.docx]

**Additional File 3**

Table a)

| **Items** |  | **Shortening of the FSS-9 (from left to right)**  **(Cronbach’ α if item deleted)** | | | | | | **Cronbach’ α if item deleted** | |
| --- | --- | --- | --- | --- | --- | --- | --- | --- | --- |
|  |  |  |  |  |  |  |  | **FSS-9 + VAFS** | **FSS-3 + VAFS** |
| **1** | 0.939 | Deleted | - | - | - | - | - | 0.939 | - |
| **2** | 0.933 | 0.933 | Deleted | - | - | - | - | 0.934 | - |
| **3** | 0.931 | 0.931 | 0.926 | 0.920 | Deleted | - | - | 0.933 | - |
| **4** | 0.932 | 0.932 | 0.926 | Deleted | - | - | - | 0.934 | - |
| **5** | 0.929 | 0.928 | 0.919 | 0.909 | 0.900 | 0.866 | **0.811** | 0.931 | 0.804 |
| **6** | 0.932 | 0.931 | 0.924 | 0.916 | 0.912 | 0.887 | **0.821** | 0.933 | 0.821 |
| **7** | 0.931 | 0.930 | 0.921 | 0.912 | 0.898 | 0.861 | **0.814** | 0.932 | 0.816 |
| **8** | 0.930 | 0.929 | 0.920 | 0.909 | 0.900 | Deleted* | - | 0.931 | - |
| **9** | 0.930 | 0.930 | 0.920 | 0.910 | 0.897 | to.869 | Deleted** | 0.932 | - |
| **VAFS** | - | - | - | - | - | - | - | 0.939 | 0.869 |
| **Cronbach’s α** | | | | | | | | **Cronbach’s α** | |
| **FSS-9** | 0.939 | 0.939 | 0.933 | 0.926 | 0.920 | 0.900 | 0.869 | 0.940 | 0.878 |

FSS-9: No. = 654, VAFS: No. = 655

Table b)

| **Items** |  | **Shortening of** **the FSS-9 (from left to right)**  **(Corrected Item-Total correlation)** | | | | | | **Corrected Item-Total correlation** | |
| --- | --- | --- | --- | --- | --- | --- | --- | --- | --- |
|  |  |  |  |  |  |  |  | **FSS-9 + VAFS** | **FSS-3 + VAFS** |
| **1** | 0.642 | Deleted | - | - | - | - | - | 0.644 | - |
| **2** | 0.756 | 0.749 | Deleted | - | - | - | - | 0.750 | - |
| **3** | 0.781 | 0.780 | 0.744 | 0.733 | Deleted | - | - | 0.782 | - |
| **4** | 0.760 | 0.754 | 0.741 | Deleted | - | - | - | 0.757 | - |
| **5** | 0.810 | 0.819 | 0.819 | 0.814 | 0.799 | 0.789 | **0.754** | 0.813 | 0.771 |
| **6** | 0.769 | 0.774 | 0.770 | 0.759 | 0.737 | 0.732 | **0.743** | 0.766 | 0.729 |
| **7** | 0.790 | 0.783 | 0.797 | 0.791 | 0.808 | 0.804 | **0.751** | 0.787 | 0.746 |
| **8** | 0.800 | 0.806 | 0.809 | 0.813 | 0.802 | Deleted* | - | 0.807 | - |
| **9** | 0.795 | 0.792 | 0.804 | 0.808 | 0.817 | 0.781 | Deleted** | 0.801 | - |
| **VAFS** | - | - | - | - | - | - | - | 0.680 | 0.635 |

FSS-9: No. = 654, VAFS: No. = 655

Table c)

| **Items** |  | **Shortening of the FSS-9** **items (from left to right)**  **(Cronbach’** **α if item deleted)** | | | | | | **Cronbach’ α if item deleted** | |
| --- | --- | --- | --- | --- | --- | --- | --- | --- | --- |
|  |  |  |  |  |  |  |  | **FSS-9 + VAFS** | **FSS-3 + VAFS** |
| **1** | 0.925 | Deleted | - | - | - | - | - | 0.927 | - |
| **2** | 0.918 | 0.919 | 0.915 | Deleted | - | - | - | 0.921 | - |
| **3** | 0.915 | 0.913 | 0.906 | 0.908 | Deleted | - | - | 0.919 | - |
| **4** | 0.919 | 0.920 | Deleted | - | - | - | - | 0.922 | - |
| **5** | 0.914 | 0.912 | 0.905 | 0.896 | 0.885 | 0.845 | **0.787** | 0.918 | 0.802 |
| **6** | 0.917 | 0.917 | 0.911 | 0.906 | 0.905 | 0.876 | **0.815** | 0.921 | 0.810 |
| **7** | 0.912 | 0.911 | 0.903 | 0.894 | 0.880 | 0.832 | **0.745** | 0.917 | 0.795 |
| **8** | 0.915 | 0.913 | 0.905 | 0.896 | 0.883 | Deleted* | - | 0.919 | - |
| **9** | 0.916 | 0.915 | 0.907 | 0.898 | 0.882 | 0.843 | Deleted** | 0.921 | - |
| **VAFS** | - | - | - | - | - | - | - | 0.925 | 0.842 |
| **Cronbach’s α** | | | | | | | | **Cronbach’s α** | |
| **FSS-9** | 0.925 | 0.925 | 0.920 | 0.915 | 0.908 | 0.883 | 0.843 | 0.928 | 0.852 |

FSS-9: No. = 224, VAFS: No. = 223

Table d)

| **Items** |  | **Shortening of the FSS-9 items (from left to right)**  **(Corrected Item-Total correlation)** | | | | | | **Corrected Item-Total correlation** | |
| --- | --- | --- | --- | --- | --- | --- | --- | --- | --- |
|  |  |  |  |  |  |  |  | **FSS-9 + VAFS** | **FSS-3 + VAFS** |
| **1** | 0.595 | Deleted | - | - | - | - | - | 0.600 | - |
| **2** | 0.705 | 0.692 | 0.672 | Deleted | - | - | - | 0.716 | - |
| **3** | 0.763 | 0.771 | 0.767 | 0.702 | Deleted | - | - | 0.766 | - |
| **4** | 0.695 | 0.685 | Deleted | - | - | - | - | 0.696 | - |
| **5** | 0.772 | 0.780 | 0.778 | 0.786 | 0.778 | 0.757 | **0.706** | 0.772 | 0.717 |
| **6** | 0.720 | 0.717 | 0.720 | 0.715 | 0.680 | 0.674 | **0.674** | 0.727 | 0.697 |
| **7** | 0.811 | 0.800 | 0.792 | 0.799 | 0.802 | 0.791 | **0.748** | 0.805 | 0.737 |
| **8** | 0.762 | 0.768 | 0.772 | 0.788 | 0.789 | Deleted* | - | 0.756 | - |
| **9** | 0.740 | 0.748 | 0.753 | 0.774 | 0.790 | 0.760 | Deleted** | 0.727 | - |
| **VAFS** | - | - | - | - | - | - | - | 0.672 | 0.642 |

FSS-9: No. = 224, VAFS: No. = 223
